# Ectopic Pancreatic Adenocarcinoma Arising in the Mesentery of the Jejunum: A Case Report

**DOI:** 10.70352/scrj.cr.26-0120

**Published:** 2026-07-17

**Authors:** Izuru Furusho, Teijiro Hirashita, Wataru Miyoshino, Hiroomi Takayama, Yoko Kawano, Yuiko Nagasawa, Shun Nakamura, Masahiro Kawamura, Takashi Masuda, Yuichi Endo, Tsutomu Daa, Masafumi Inomata

**Affiliations:** 1 Department of Gastroenterological and Pediatric Surgery, Oita University Faculty of Medicine, Yufu, Oita, Japan; 2 Department of Diagnostic Pathology, Oita University Faculty of Medicine, Yufu, Oita, Japan

**Keywords:** ectopic pancreas, aberrant pancreas, ectopic pancreatic cancer, mesentery

## Abstract

**INTRODUCTION:**

Ectopic pancreas is defined as pancreatic tissue lacking anatomical and vascular continuity with the main pancreas. Malignant transformation within ectopic pancreatic tissue is extremely rare, and cases arising in the mesentery are exceptionally uncommon. We report a rare case of adenocarcinoma arising from ectopic pancreatic tissue in the proximal jejunal mesentery.

**CASE PRESENTATION:**

An 80-year-old man presented with abdominal pain. Contrast-enhanced CT showed a 3-cm mass adjacent to the proximal jejunum, initially suspected to be a jejunal submucosal tumor, such as gastrointestinal stromal tumor. Laparoscopy identified a firm mesenteric tumor closely associated with the superior mesenteric artery and jejunal branches, necessitating conversion to open surgery. The tumor was completely excised while preserving the small intestine. Histopathological examination demonstrated well-differentiated adenocarcinoma arising within ectopic pancreatic tissue containing acinar, ductal, and islet of Langerhans. Immunohistochemistry showed positive staining for carbohydrate antigen 19-9, p16, p53, and S100A4, supporting the diagnosis of pancreatic ductal adenocarcinoma. The postoperative course was uneventful except for transient paralytic ileus. The patient was discharged on POD 19 and has remained recurrence-free for 9 months.

**CONCLUSIONS:**

Adenocarcinoma arising from ectopic pancreatic tissue in the mesentery is exceedingly rare and difficult to diagnose preoperatively. Complete surgical excision is essential for definitive diagnosis and curative treatment. Further case accumulation is required to better define its clinical characteristics and optimal treatment strategies.

## Abbreviations

CA19-9
: carbohydrate antigen 19-9
CEA
: carcinoembryonic antigen
GIST
: gastrointestinal stromal tumor
SMA
: superior mesenteric artery

## INTRODUCTION

Ectopic pancreas is defined as pancreatic tissue that exists outside the normal pancreas and lacks anatomical and vascular continuity with it. The reported incidence in autopsy studies ranges from 0.5% to 13.7%.^1)^ Common sites include the stomach, duodenum, and jejunum, whereas occurrence within the mesentery is extremely rare.^2)^ Malignant transformation of ectopic pancreas is exceedingly uncommon. Here, we report a rare case of adenocarcinoma arising from ectopic pancreas in the mesentery of the proximal jejunum.

## CASE PRESENTATION

An 80-year-old man presented to a previous hospital with abdominal pain. Abdominal CT revealed a 3-cm mass in the left upper quadrant. A tumor arising from the duodenum or proximal small intestine was suspected, and the patient was referred to our department for further evaluation and treatment. Although the abdominal pain led to the initial detection, the lesion was considered unlikely to be the cause, and the etiology of the pain remained unclear. His past medical history included appendectomy at 50 years of age, and his family history was notable only for gastric cancer in his father. Laboratory findings were unremarkable. Serum CEA was within the normal range (3.76 ng/mL), whereas CA19-9 was slightly elevated at 44.96 U/mL (reference range, ≤37.0 U/mL). CT was performed with non-contrast and equilibrium-phase imaging, using a slice thickness of 1 mm. It demonstrated a 3-cm mass located at the fourth portion of the duodenum and the proximal jejunum, adjacent to the SMA and its jejunal branches, without lymph node enlargement or distant metastases, and showing enhancement in the equilibrium phase (**Fig. 1**). Upper gastrointestinal endoscopy revealed no mucosal abnormalities in the duodenum and proximal jejunum. Based on these findings, the lesion was preoperatively diagnosed as a jejunal submucosal tumor, such as GIST, and surgical resection was planned. Laparoscopic exploration revealed a firm, immobile mass within the mesentery of the proximal jejunum (**Fig. 2A** and **2B**). Because of its proximity to the SMA and limited mobility, the procedure was converted to an open approach. The tumor was located on the dorsal side of the mesentery and was carefully dissected from the SMA, with ligation of the first jejunal vessels and preservation of the second jejunal artery. The tumor was completely excised, and the jejunal blood flow remained adequate, allowing preservation of the jejunum because the marginal vessels of the jejunum could be preserved (**Fig. 2C** and **2D**). Based on the preoperative diagnosis, lymph node dissection was not performed. The resected specimen measured 34 mm and showed a solid, whitish appearance on the cut surface (**Fig. 3**). Histopathological examination revealed well-differentiated adenocarcinoma forming glandular structures within fibrous stroma (**Fig. 4A**–**4C**). Non-neoplastic pancreatic tissue, including islets of Langerhans, was identified adjacent to the carcinoma (**Fig 4A**), and residual islets of Langerhans were observed within the tumor (**Fig. 4D**). These findings suggested that the carcinoma arose at the site of the ectopic pancreas. Immunohistochemical staining demonstrated positivity for CA19-9, p16, p53, and S100A4, negativity for SMAD4 and DUPAN-2, and lymphovascular invasion detected by D2-40 staining (**Fig. 5A**–**5D** and **Supplementary Table 1**). The loss of SMAD4 expression and positivity for CA19-9 supported a pancreatic ductal origin of the tumor. The lymphatic invasion was localized and did not extend to the resection margin. These findings confirmed the diagnosis of well-differentiated adenocarcinoma arising from ectopic pancreatic tissue. Surgical margins were negative. According to the TNM/AJCC (8th edition) classification for pancreatic cancer, the pathological stage was T3NxM0. Postoperatively, the patient developed transient paralytic ileus, which resolved with conservative management. He was discharged on POD 19. Adjuvant chemotherapy, in accordance with standard management for pancreatic cancer, was recommended; however, the patient declined. Nine months after surgery, the patient remains recurrence-free under outpatient follow-up.

**Fig. 1:**
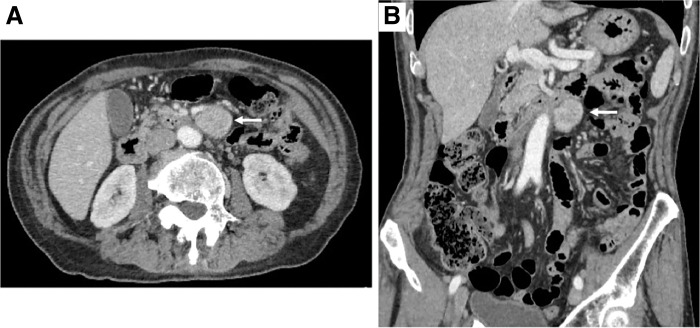
(**A**, **B**) Contrast-enhanced CT. A 3-cm mass (arrows) is located at the junction of the fourth portion of the duodenum and the proximal jejunum, adjacent to the SMA and its jejunal branches. SMA, superior mesenteric artery

**Fig. 2:**
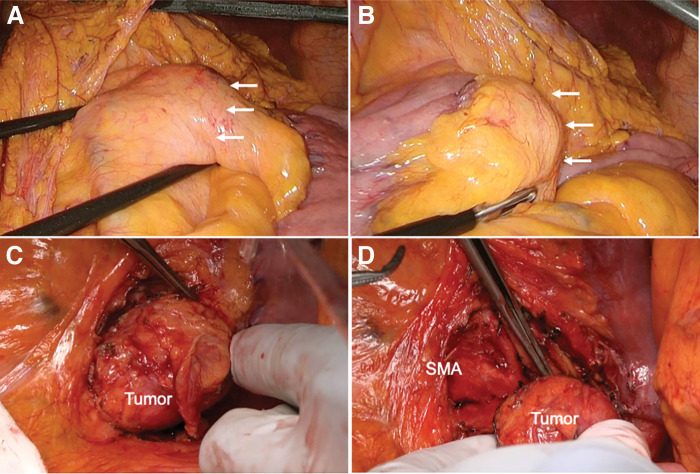
Operative findings. Laparoscopic ventral (**A**) and dorsal (**B**) views of the tumor (arrows) in the mesentery. The tumor was located on the dorsal side of the mesentery (**C**) and was carefully dissected from the SMA (**D**). SMA, superior mesenteric artery

**Fig. 3:**
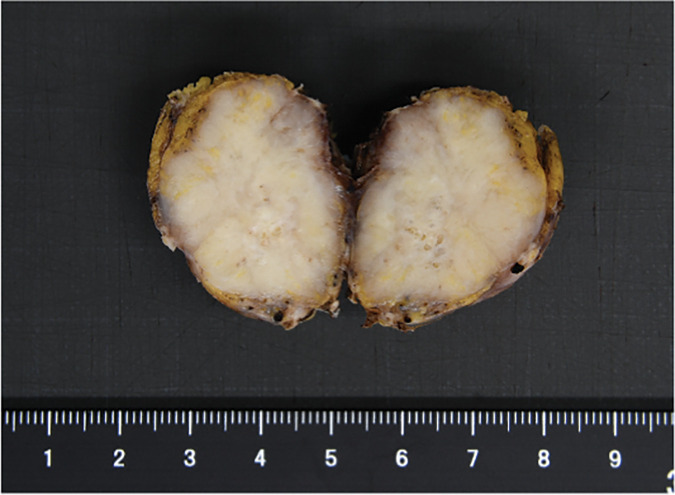
Resected specimen. The resected specimen showed that the tumor measured 34 mm and was solid and whitish on the cut surface.

**Fig. 4:**
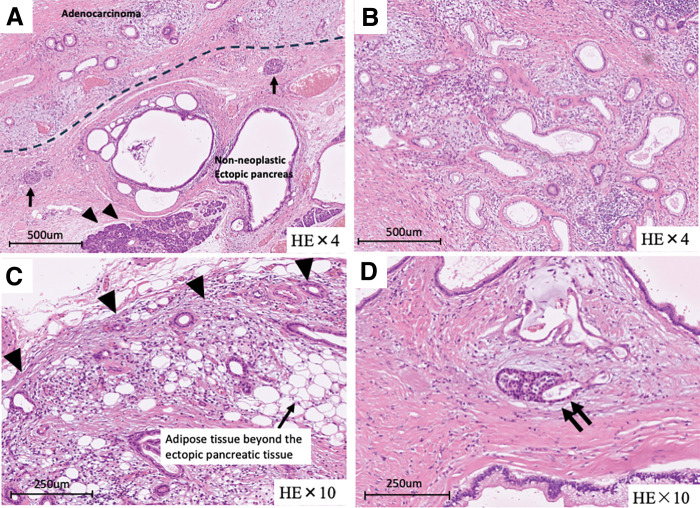
Pathological findings. (**A**) Neoplastic glandular structures are observed above the dashed line, corresponding to the adenocarcinoma component. Below the dashed line, ectopic pancreatic tissue (arrowheads) with islets of Langerhans (arrows) is identified, and the 2 components are in close proximity. (**B**) Tumor cells were proliferating with accompanying fibrous stroma. (**C**) Infiltration of tumor cells into the surrounding stroma was observed. Arrowheads show the invasive front of the tumor. (**D**) Residual islets of Langerhans (arrows) entrapped within the tumor tissue. HE, hematoxylin and eosin

**Fig. 5:**
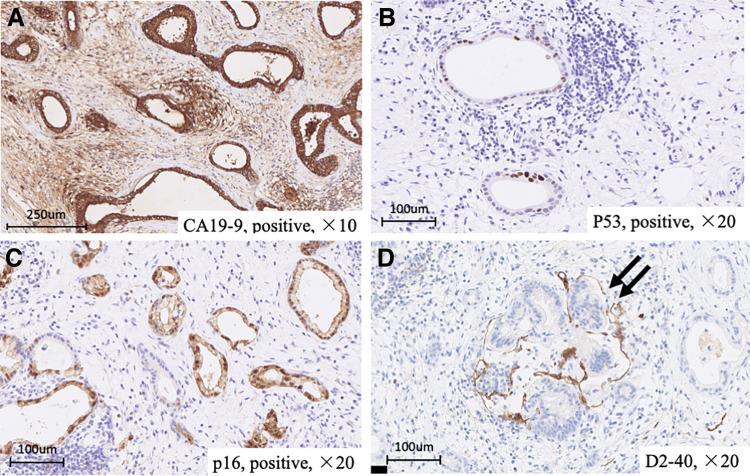
Immunohistochemical findings. Representative immunohistochemical staining images of CA19-9 (**A**, positive, ×10), p53 (**B**, positive, ×20), and p16 (**C**, positive, ×20). D2-40 staining (**D**, ×20) highlights lymphatic vessels, and arrows indicate lymphatic invasion by tumor cells. Scale bars: 250 μm in (**A**) and 100 μm in (**B**–**D**). CA19-9, carbohydrate antigen 19-9

## DISCUSSION

Ectopic pancreas is considered to arise from aberrant migration of pancreatic tissue during embryogenesis, possibly due to incomplete fusion of the dorsal and ventral pancreas. Consequently, it most commonly occurs in regions adjacent to the pancreas, particularly in the stomach (30%), duodenum (30%), and jejunum (20%),^3)^ whereas involvement of the mesentery is exceedingly rare.^4,5)^ Reported cases show a male predominance, with a male-to-female ratio of approximately 2:1.^6)^

Histopathological classification of ectopic pancreas and the diagnostic criteria for carcinoma arising from it have been described in previous studies. Von Heinrich categorized ectopic pancreas into 3 histologic types^7)^: Type I, containing all pancreatic elements (acini, ducts, and islets); Type II, composed of acini and ducts without islets; and Type III, consisting only of ducts surrounded by smooth muscle. Guillou et al.^8)^ proposed 3 criteria for diagnosing carcinoma arising within ectopic pancreas: (1) the carcinoma must be located close to non-neoplastic pancreatic tissue; (2) pancreatic acini or islets must be present within the lesion, and (3) there must be a direct transition between pancreatic structures and carcinoma. In our case, the presence of islets indicated Type I ectopic pancreas, and the first 2 of Guillou’s criteria were clearly fulfilled, and the close association between the ectopic pancreatic tissue and carcinoma strongly suggested a pancreatic origin. Furthermore, immunohistochemically, the tumor cells were positive for CA19-9, while SMAD4 expression was lost, a finding characteristic of pancreatic ductal adenocarcinoma and reported in approximately 60% of pancreatic cancers.^9)^ Collectively, these findings favored the diagnosis of ectopic pancreatic carcinoma.

Preoperative diagnosis of ectopic pancreatic carcinoma is challenging because of its rarity and nonspecific imaging features. In our case, the lesion was preoperatively diagnosed as a jejunal submucosal tumor, such as GIST. Similar lesions have frequently been misinterpreted as GISTs, lymphomas, or metastatic tumors from gastrointestinal malignancies.^10,11)^ Although the presence of a central duct-like structure on imaging may suggest ectopic pancreas,^12)^ such findings may disappear once malignant transformation occurs. Slight preoperative elevation of CA19-9, as observed in our patient, may provide a diagnostic clue; Yoshida et al. reported CA19-9 elevation in 7 of 11 cases with carcinoma of the jejunal ectopic pancreas.^13)^

A PubMed search identified several case reports and limited reviews on ectopic pancreatic adenocarcinoma. Cazacu et al.^14)^ reported that malignant transformation of ectopic pancreas is rare (0.7%–1.8%) and difficult to diagnose due to nonspecific clinical and radiological features, with the stomach being the most common site, followed by the duodenum and jejunum. Recent reports have emphasized the diagnostic challenges associated with malignant ectopic pancreas because of its nonspecific clinical presentation and frequent manifestation as gastrointestinal obstruction.^15,16)^ Histologically, adenocarcinoma is the predominant type, and preoperative diagnosis is often challenging, making surgical resection essential for both diagnosis and treatment. Jejunal involvement is particularly rare; Yamaoka et al.^17)^ identified only 7 reported cases of jejunal ectopic pancreatic carcinoma, all of which were diagnosed after surgical resection and frequently presented with bowel obstruction. Small bowel intussusception has also been reported as an initial manifestation of jejunal ectopic pancreatic carcinoma, further underscoring the nonspecific clinical presentation and diagnostic challenges associated with this rare disease.^18)^ More recently, Eizuka et al.^19)^ reviewed the literature published between 1995 and 2025 and identified only 8 cases, including their own, underscoring the extreme rarity and diagnostic challenges associated with jejunal ectopic pancreatic carcinoma. Although some reports suggest a more favorable outcome than that of primary pancreatic ductal adenocarcinoma, the overall prognosis remains poor, with reported mortality rates of approximately 14% at 1 year and 25% at 3 years.^15)^ Jejunal cases may have an even worse prognosis, with frequent distant metastasis and short survival in advanced disease.^17)^ Optimal management strategies, including lymphadenectomy and chemotherapy, have not been established; thus, surgical resection is currently considered the only reliable treatment option. To the best of our knowledge, ectopic pancreatic adenocarcinoma arising in the small intestinal mesentery has not been previously reported in the English-language literature.

Complete surgical resection with negative margins (R0 resection) is essential for localized pancreatic cancer. The role of lymph node dissection remains unclear. Hoshino et al. reviewed 20 reported cases of jejunal ectopic pancreatic carcinoma and found lymph node or distant metastasis in 8 cases and recurrence within 1 year in another 8, indicating poor prognosis.^20)^ Therefore, adjuvant chemotherapy following the standard regimen for pancreatic cancer is commonly employed. However, the patient declined the postoperative S-1 therapy. During the first 2 years, serum CA19-9 levels will be measured every 3 months and contrast-enhanced CT will be performed every 6 months. Thereafter, follow-up will be conducted at 6-month intervals.

This case highlights the importance of including carcinoma arising from ectopic pancreas in the differential diagnosis of mesenteric tumors and underscores the role of complete surgical resection and detailed pathological evaluation in achieving accurate diagnosis and appropriate management.

## CONCLUSIONS

We report an extremely rare case of adenocarcinoma arising from ectopic pancreas located in the jejunal mesentery. Preoperative diagnosis is difficult because of its rarity and nonspecific imaging findings. Complete surgical resection is indispensable for both definitive diagnosis and curative treatment. Further accumulation of similar cases is necessary to establish optimal management strategies for this uncommon entity.

## SUPPLEMENTARY MATERIALS

Supplementary Table 1 Details of immunohistochemical staining.

## DECLARATIONS

### Availability of data and materials

The data that support the findings of this study are available from the corresponding author upon reasonable request.
