# Supplementary material for: Ectopic Pancreatic Adenocarcinoma Arising in the Mesentery of the Jejunum: A Case Report
Source: Surg Case Rep. 2026 Jul 17;12(1):26-0120. doi: 10.70352/scrj.cr.26-0120 (PMC13384810; doi:10.70352/scrj.cr.26-0120)
Supplement: Supplementary Table 1 — Details of immunohistochemical staining. [file scr-12-01-26-0120-s001.pdf]

Supplementary Table 1 Details of immunohistochemical staining

Marker	Clone	Dilution	Result	Extent/intensity
CA19-9	C241:5:1:4 (Leica)	1:200	Positive	Diffuse/Strong
p16	6H12 (Leica)	Prediluted	Positive	Focal/Strong
p53	DO-7 (Leica)	Prediluted	Positive	Focal/Strong
S100A4	922813 (R6D Systems)	1:100	Positive	Diffuse/Strong
SMAD4	B-8 (Santa Cruz Biotechnology)	1:10	Negative	(-) / not applicable
DUPAN-2	DU-PAN-2 (Hitachi Chemical Diagnostics Systems)	1:100	Negative	(-) / not applicable
D2-40	D2-40 (Roche)	1:100	Positive	Focal/Strong
